# Prenatally diagnosed congenital pyloric atresia in consecutive three siblings: a case report

**DOI:** 10.1186/s40792-020-01096-1

**Published:** 2021-01-06

**Authors:** Ryuta Saka, Dan Yamamoto, Seika Kuroda, Souji Ibuka, Tasuku Kodama, Toshimichi Hasegawa

**Affiliations:** 1 Department of Pediatric Surgery, National Hospital Organization Fukuyama Medical Center, 4-14-17 Okinogamicho, Fukuyama, Hiroshima 720-8520 Japan; 2 Department of Obstetrics and Gynecology, National Hospital Organization Fukuyama Medical Center, 4-14-17 Okinogamicho, Fukuyama, Hiroshima 720-8520 Japan; 3 Department of Pediatric Surgery, Tottori University, Tottori, Japan

**Keywords:** Congenital pyloric atresia, Prenatal diagnosis, Familial

## Abstract

**Background:**

Congenital pyloric atresia (CPA) is a rare gastrointestinal anomaly frequently associated with epidermolysis bullosa (EB). Although the complications of familial isolated CPA are minor, delays in diagnosis can increase the chances of morbidity.

**Case presentation:**

Three female infants born to a Japanese mother presented with CPA at birth. There was no consanguinity between the parents, and the spacing between pregnancies was 2 years in each case. All 3 siblings had a prenatal diagnosis of CPA owing to polyhydramnios and a dilated stomach, without dilatation of the rest of the gastrointestinal tract. All patients underwent reconstructive surgeries for establishing bowel continuity (Case 1, pyloromyotomy; Case 2, gastroduodenostomy in a diamond fashion; and Case 3, gastroduodenostomy in a side-to-side fashion) soon after birth. Their postoperative courses were uneventful, and they grew up healthily, without any complications.

**Conclusion:**

Fetal ultrasonography is useful for diagnosing CPA prenatally. Successful prenatal diagnosis can lead to timely intervention after birth.

## Background

Congenital pyloric atresia (CPA) is a rare gastrointestinal anomaly that accounts for less than 1% of all intestinal atresias [1]. Although it is easy to diagnose CPA when characteristic symptoms such as non-bilious emesis and distention of the upper abdomen soon after birth are present, delays in diagnosis may lead to serious complications, including gastric perforation and aspiration pneumonia [2]. Therefore, the role of prenatal diagnosis is important in CPA.

Anatomically, CPA can be classified into 3 subtypes: type A, pyloric membrane or web; type B, pylorus replaced by solid tissue; and type C, complete interruption with a gap between the stomach and duodenum [3]. CPA can also be classified into “isolated” and “complicated” (43–65%) clinically [4, 5]. Among the associated anomalies, epidermolysis bullosa (EB) is the most common. Although familial incidence of CPA associated with EB (autosomal recessive inheritance) has been widely documented, familial isolated CPA is rare [2, 6, 7]. Herein, we report the cases of 3 siblings who were prenatally diagnosed with isolated CPA.

## Case presentation

The patients’ parents were Japanese, and there was no consanguinity between them. They had no family history of genetic disease. A summary of the 3 cases is shown in Table 1.

**Table 1:** Summary of the clinical course of the three cases

	Case 1	Case 2	Case 3
Prenatal ultrasonography			
Polyhydramnios	+	+	+
Dilated stomach	+	+	+
Snowflake sign	–	–	–
Other anomaly	–	–	–
Time of diagnosis (WGA)	32	31	30
Delivery	VD	CS	CS
Gestational age (weeks)	34	36	36
Birthweight (g)	2114 (+ 0.4 SD)	2204 (− 1.1 SD)	2276 (− 0.8 SD)
Apgar score (1 min/5 min)	8/9	8/9	8/9
Associated anomaly	–	–	–
Operation	Pyloroplasty	GD	GD
	H-M	Diamond	Side-to-side
Time to oral full feeding (days)	18	9	14
Complications	–	–	–
Follow-up (months)	135	111	79

*WGA* weeks of gestational age, *VD* vaginal delivery, *CS* cesarean section, *SD* standard deviation; *GD* gastroduodenostomy; *H-M* Heineke–Mikulicz

### Case 1

**Fig. 1:**
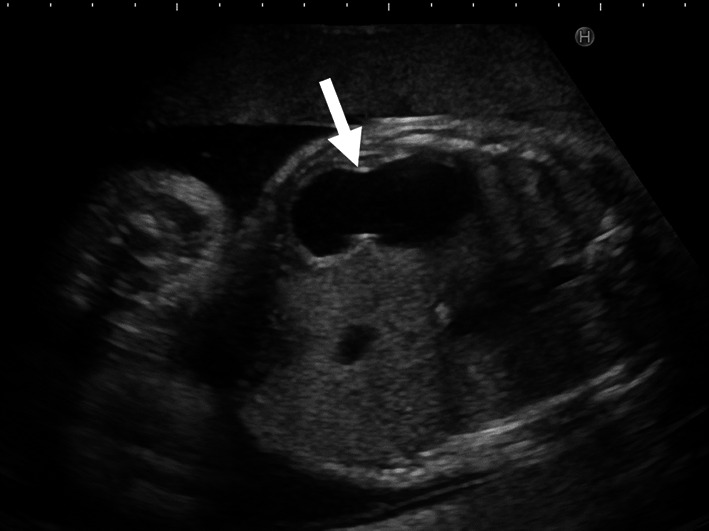
Prenatal ultrasonography revealing polyhydramnios and dilated stomach (*arrow*) (Case 1)

**Fig. 2:**
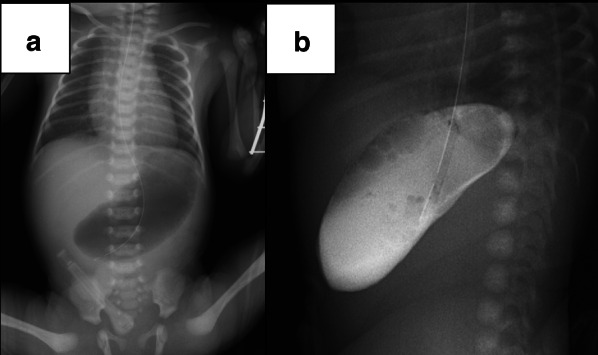
Radiological findings of Case 1: **a** plain radiograph showing dilated stomach and absence of gas in the duodenum, distally; **b** upper gastrointestinal contrast study showing dilated stomach and complete gastric outlet obstruction

A healthy, 21-year-old G1P0 woman was referred to our hospital for preterm rupture of membranes at 32 weeks of gestation. An ultrasonography on admission revealed polyhydramnios ((amniotic fluid index [AFI]: 30 cm) and a remarkably dilated fetal stomach (66 × 31 mm) (Fig. 1). However, neither an enlarged duodenum nor echogenic amniotic fluid was noted. A prenatal diagnosis of CPA was made. A girl was born by vaginal delivery at 34 weeks of gestation. Immediately after birth, a nasogastric tube was placed, and non-bilious fluid was aspirated. Plain abdominal radiography showed the “single bubble” sign (Fig. 2a), and an upper gastrointestinal contrast study showed complete obstruction of the pylorus (Fig. 2b).

**Fig. 3:**
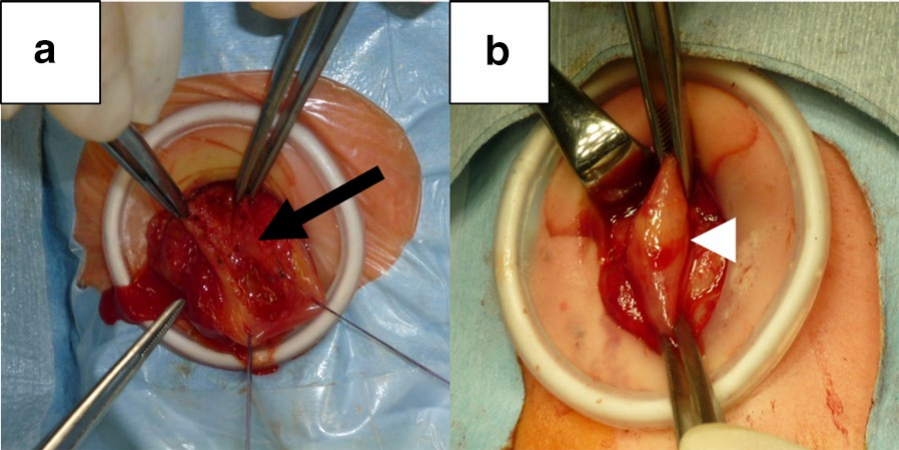
Intraoperative findings. **a** A longitudinal incision revealing that the pylorus is replaced by solid tissue (*arrow*) in Case 1. **b** The pylorus (*arrowhead*) showing continuity between the stomach and duodenum in Case 2

Laparotomy revealed a slightly yellowish and hard pylorus. The anterior wall from the stomach to the duodenum was longitudinally opened, and the atretic segment was confirmed to be a 1-cm-long segment of solid tissue (type B) (Fig. 3a). Pyloroplasty (Heineke–Mikulicz) was performed to achieve a tension-free anastomosis and to establish sufficient lumen. A nasojejunal tube was inserted for postoperative enteral feeding, and intestinal patency was confirmed with saline injection. Her postoperative course was uneventful, except neonatal apnea, which resolved spontaneously by 2 weeks of her life. She also achieved full oral intake 18 days postoperatively. She is now a healthy 11-year-old with normal growth and development.

### Case 2

**Fig. 4:**
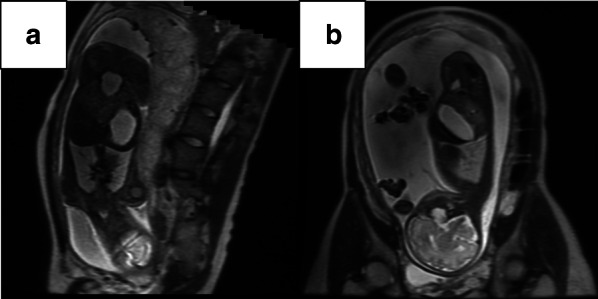
Fetal MRI showing polyhydramnios and dilated stomach in Case 2: **a** axial view, **b** sagittal view

The second pregnancy, 2 years after Case 1, was complicated by threatened premature labor, and the mother was referred to our hospital at 31 weeks of gestation. Prenatal ultrasonography revealed polyhydramnios (AFI: 27 cm) and dilatation of the fetal stomach (50 × 27 mm). Although fetal magnetic resonance imaging (MRI) showed a dilated stomach, neither dilated intestines nor an associated anomaly was confirmed (Fig. 4). A prenatal diagnosis of familial CPA was established based on the radiological findings and family history. The second sibling, a female, was delivered by cesarean section due to maternal indication at 36 weeks of gestation. Following decompression of the stomach, plain abdominal radiography and an upper gastrointestinal contrast study confirmed the diagnosis of CPA.

Laparotomy confirmed type B CPA, and the atretic segment was 1 cm in length (Fig. 3b). Bowel continuity was restored by gastroduodenostomy in a diamond fashion (transverse gastrotomy in the stomach and longitudinal duodenotomy) without excision of the atretic segment. A nasojejunal tube was inserted, and patency of the intestine was confirmed with saline injection. Her postoperative course was uneventful, and she achieved full oral intake 9 days postoperatively. She is now a healthy 9-year-old with normal growth and development.

### Case 3

The third pregnancy, 2 years after Case 2, was again complicated by threatened premature labor. Prenatal ultrasonography showed polyhydramnios (AFI: 27 cm) and a dilated fetal stomach (45 × 16 mm) at 30 weeks of gestation. The third sibling, who was also female, was delivered by cesarean section at 36 weeks of gestation. Plain abdominal radiography and an upper gastrointestinal contrast study revealed complete obstruction of the pylorus, leading to the diagnosis of CPA.

On laparotomy, type B CPA (atretic segment: 1 cm in length) was confirmed. In this case, gastroduodenostomy was performed in a side-to-side fashion without excision of the atretic segment. A nasojejunal tube was placed, and patency of the intestines was confirmed. Her postoperative course was uneventful. She achieved full oral intake 14 days postoperatively. She is now a healthy 6-year-old with normal growth and development.

## Discussion

Congenital pyloric atresia (CPA) is an extremely rare gastrointestinal anomaly that accounts for less than 1% of all gastrointestinal atresias, with an incidence of 1 per 100,000 live births [1]. CPA can be classified into 3 anatomical types: type A, which shows a pyloric membrane or web, which can be multiple (57%); type B, in which the pyloric channel is replaced by a solid cord (34%); and type C, which shows complete interruption between the stomach and the duodenum (9%) [3]. In our cases, all 3 siblings showed type B CPA.

Type B CPA is treated with either pyloroplasty (Heineke–Mikulicz or Finney) or gastroduodenostomy (end-to-end, side-to-side, and diamond-shaped), with or without excision of the atretic segment [4, 5, 8]. Gastrojejunostomy should be avoided due to high morbidity, including anastomotic ulcers [9]. Regardless of how it is adapted, patency of the remaining intestine should be confirmed intraoperatively to exclude the possibility of associated intestinal atresia. Dessanti et al. reported two cases of type B CPA treated with their novel approach, gastroduodenal mucosal advancement anastomosis, for preservation of pyloric function [10]. The surgical procedure can be chosen on the bases of the length of the atretic portion, positions of the stomach and duodenum, and the surgeons’ experience. Although 3 different methods were used based on surgeons’ preference and experience in our series, the atretic segment was not completely excised in any case. All 3 girls are healthy, and of normal height and weight in the relatively long observational period (79 – 134 months). They neither have epigastric pain nor have problems with gastric emptying. Complete excision of the atretic segment may not be essential due to the potential complication of hepatoduodenal ligament damage.

CPA is frequently associated with other anomalies (43–66%), including epidermolysis bullosa (EB) [4, 5, 8]. CPA can also be a part of hereditary multiple intestinal atresias, which show high mortality and morbidity. Although the overall mortality of CPA is very high, isolated CPA shows good prognosis [5]. The prognosis depends on the associated anomalies. Familial occurrence of CPA, especially with EB, has been well documented, and approximately one-third of patients in a large case series had familial CPA [4, 5]. Usui et al. reviewed familial isolated CPA and reported 15 families (34 individuals) [2]. Although some gene mutations (*ITGA6*, *ITGB4*, and *PLEC*) are known to cause EB with CPA, little is known about gene mutations in isolated CPA [11]. In our series, genetic testing was not desired by the parents.

Without prenatal diagnosis, accurate diagnosis after birth might be delayed because of non-bilious emesis. Complications resulting from delays in diagnosis (including aspiration pneumonia and gastric perforation) can be fatal, regardless of the excellent prognosis of isolated CPA [2, 9]. Therefore, the role of prenatal diagnosis is important even in isolated CPA. CPA can be diagnosed prenatally by ultrasonography with the findings of polyhydramnios, enlarged fetal stomach, and the absence of dilated intestine, distally [7, 12]. Prenatal diagnosis of CPA was made between 30 and 32 weeks of gestation in all three of our cases. Goldstein et al. reported that fetal stomach growth is closely correlated with gestational age, and that normal fetal stomach at 31 to 33 weeks of gestation is 2.8 ± 0.9 cm in size, longitudinally [13]. Dilatation of the stomach should be evaluated according to the gestational age. The fetal stomach was dilated according to the gestational age throughout the observation period in this series. In cases of CPA complicated with EB, echogenic amniotic fluid (“snowflake sign”), dysplastic external ear and nose, and complete chorioamniotic membrane separation can be observed with ultrasonography, along with skin blistering [14, 15]. In our series, such findings were not noted throughout the course of the 3 pregnancies. Fetal MRI, which shows an enlarged stomach, polyhydramnios, and dilated esophagus in CPA, may also be helpful for the diagnosis of associated anomalies including EB [16, 17]. In Case 2, fetal MRI was performed to confirm the prenatal diagnosis, because this case was our first experience of familial isolated CPA.

## Conclusions

We herein report 3 cases of familial isolated CPA in siblings in which fetal ultrasonography was useful for diagnosing CPA prenatally. Prenatal diagnosis can lead to timely intervention after birth in such cases.

## Data Availability

The authors declare that all data in this manuscript are available within the article.

## Abbreviations

CPA: Congenital pyloric atresia
EB: Epidermolysis bullosa
MRI: Magnetic resonance imaging
AFI: Amniotic fluid index
